# Detection of activating mutations in liquid biopsy of Egyptian breast cancer patients using targeted next-generation sequencing: a pilot study

**DOI:** 10.1186/s43046-021-00067-3

**Published:** 2021-04-17

**Authors:** Neemat Kassem, Hebatallah Kassem, Loay Kassem, Mohamed Hassan

**Affiliations:** 1 Clinical and Chemical Pathology Department, Kasr Al Ainy Centre of Clinical Oncology & Nuclear Medicine, School of Medicine, Cairo University, Cairo, Egypt; 2 Clinical Oncology Department, School of Medicine, Cairo University, Cairo, Egypt

**Keywords:** Breast cancer, Next-generation sequencing, Activating mutations, Microsatellite instability

## Abstract

**Background:**

Breast cancer (BC) is the 2^nd^ most prevalent malignancy worldwide and is the most prevalent cancer among Egyptian women. The number of newly described cancer-associated genes has grown exponentially since the emergence of next-generation sequencing (NGS) technology. We aim to identify activating mutations in liquid biopsy of Egyptian breast cancer patients using targeted NGS technology. We also demonstrate the microsatellite instability (MSI) status using BAT25, BAT26, and NR27 markers which are tested on the Bioanalyzer 2100 system.

**Results:**

Twenty-one variants were detected in 15 genes: 7 Substitution-Missense, 12 Substitution-coding silent, and 2 Substitution-intronic. Regarding ClinVar database, out of 21 variants there were 14 benign variants, 3 variants with conflicting interpretations of pathogenicity, 3 variants not reported, and 1 drug response variant. *TP53* p.(Pro72Arg) missense mutations were found in 75% of patients. *PIK3CA* p.(Ile391Met), *KDR* p.(Gln472His) missense mutations were detected in 25% of patients each. Two patients revealed APC gene missense mutation with p.(Ile1307Lys) and p.(Glu1317Gln) variants. Only one patient showed *ATM* p.(Phe858Leu) gene mutation and one showed FGFR3 p.(Ala719Thr) variant. Regarding microsatellite instability (MSI) status, 2/8 (25%) patients were MSS, 3/8 (37.5%) patients were MSI-L, and 3/8 (37.5%) patients were MSI-HI.

**Conclusion:**

It is essential to use and validate minimally invasive liquid biopsy for activating mutations detection by next-generation sequencing especially in patients with inoperable disease or bone metastasis. This work should be extended with larger patient series with comparison of genetic mutations in liquid-based versus tissue-based biopsy and longer follow up period.

## Background

Breast cancer (BC) is the 2^nd^ most prevalent and lethal malignancy worldwide and is the most prevalent cancer among Egyptian women [1]. In Egypt, National Cancer Registry Program (NCRP) revealed that the commonest cancer sites were liver, breast, and bladder (23.8%, 15.4 and 6.9%, respectively) in both genders; liver and bladder (33.6% and 10.7%, respectively) in men; and breast and liver (32% and 13.5%, respectively) in females [2]. Bone is the most common location of BC metastasis; these metastases are found in 65–75% of metastatic BC patients [3]. Furthermore, bone has been noted to be the most common location of first distant BC relapse [4].

Decades of research has generated the recognition that cancer is a genetic disorder, revealing that it is the accumulation of molecular alternations which is the principle factor of tumorigenesis, guiding the acquisition of the malignant phenotype [5]. The number of newly described cancer-associated genes has grown exponentially since the emergence of next-generation sequencing (NGS) technology [6].

For many decades, the single available substance for molecular testing was the patient’s formalin-fixed paraffin-embedded (FFPE) tumor tissue. This FFPE samples have many advantages as it is accessible material, simple for usage and storage. Also, it gives the chance to choose appropriate tumor tissue, elevating the sensitivity of genetic mutation detection assays [7]. On the other hand, FFPE material has obvious disadvantages, such as inability to acquire in cases of inoperable tumors and bone metastases with some difficulties to capture the tumor’s heterogeneity. Moreover, this genetic material collected as a result of paraffin processing of tissue, is commonly of low/poor quality, and not adequate for molecular profiling [8]. The most essential point that the molecular profile of cancer is transformed, basically after targeted therapy and these alternations cannot be noticed by testing the 1ry tumor material but require invasive tissue rebiopsies [9].

The presence of neoplastic features in the plasma DNA of cancer patients was first observed in 1989 [10]. Later on, several reports have found that testing of cell-free tumor-derivative nucleic acids in cancer patient’s body fluids (serum, plasma, urine, stool, bronchoalveolar lavage, etc.) may be used to determine tumor specific variations [11]. The word liquid biopsy has came up demonstrating the use of those minimally invasive materials for tumor characterization. The mutations noticed in liquid biopsies reflect mutations found in the patient’s tumor itself. As well as, circulating tumor nucleic acids (ctNA) analysis could eventually determine more genetic alternations compared to analysis of a particular area in a FFPE tumor tissue, as it arises from the whole tumor’s area and/or metastasis present in the patient’s body, so being characteristic of intra and inter-tumor heterogeneity [12]. The usage of plasma samples for ctNA analysis has recently become attainable because of the improvement of sensitive molecular techniques that can determine with high accuracy minimal amounts of ctNAs that are present in these liquid biopsies. For this intend, several techniques have been used, such as digital PCR, real-time PCR, Arms PCR, and NGS [13].

In this study, we aim to identify activating mutations in liquid biopsy of Egyptian breast cancer patients using targeted next-generation sequencing technology. We also demonstrate the microsatellite instability (MSI) status using BAT25, BAT26, and NR27 markers which are tested on the Bioanalyzer 2100 system.

## Methods

### Study population

The study included 8 Egyptian breast cancer patients who are attending to outpatient clinic of our department. Patients were selected according to the following inclusion criteria: adult, females, and confirmed pathological examination of invasive carcinoma of no specific type. Seven patients received neoadjuvant chemotherapy and underwent surgical interventions. After that, each patient was treated individually and received either adjuvant chemotherapy or hormonal treatment according to international guidelines. At this stage, blood samples were withdrawn from those patients under strict sterile conditions for molecular testing. Clinico-pathological features at diagnosis were collected from patients’ records. This study was done in the molecular Lab. of our institution, in the period from December 2019 to June 2020. The study was approved by Institutional Review Board (IRB)-11-2019 of our clinical oncology department. All procedures carried out in the study including human participants were in agreement with the ethical standards of the institutional research committee and with the 1964 Helsinki declaration and its later amendments (GCP guidelines) or comparable ethical standards.

### DNA extraction

According to the manufacturer protocol, genomic DNA (gDNA) was extracted from those liquid biopsies with the QIAamp DNA Mini kit (QIAGEN, Germany, Cat No./ID: 51304) and was eluted in 60 μL volume. Concentration of extracted DNA samples was measured by the Qubit dsDNA High Sensitivity (HS) assay kit (Life Technologies-Fisher Scientific, Cat No.:Q32851). Testing quality and amplifiability of the extracted gDNA samples was done by quantitative PCR (qPCR) technology. All samples with Δ Cq value below 5 can be selected for further use.

### Library preparation and sequencing

Following the manufacturer instructions, the libraries were prepared using AmpliSeq™ for Illumina Cancer Hotspot Panel v2 (Illumina, Inc., US, Cat. No.: 20019161) which is a targeted next generation sequencing assay detecting actionable mutations across the hotspot regions of 50 genes. Quality of the libraries was checked out by Agilent 2100 Bioanalyzer device utilizing the DNA 1000 reagents and Chips (Agilent Technologies, Santa Clara, California, Cat. Code: 5067-1504). The expected PCR product is 186–277 bp, which indicates successful library amplification. Patients’ libraries together with PhiX control library were normalized and equal volumes were pooled to form the terminal sequencing library. The AmpliSeq™ for Illumina Cancer Hotspot Panel achieves detection limits of 5% variant allele frequency across 207 amplicons with > 95% of bases covered at ≥ 500× [14]. Sequencing was done using Cancer Hotspot Panel v2 Nano kit on MiSeqDx device (llumina) with a 2 × 150 bp read length and total time of ~ 17 h which involve cluster generation, sequencing, and base calling on the MiSeqDx system.

### Bioinformatics and data analysis

Bioinformatics and data analysis start from checking each run quality through assessing the specifications based on Illumina PhiX control library which support cluster densities between 865–965 k/mm^2^ clusters passing filter for v2 chemistry. The second item is the quality score (Q-score) which is a prediction of the probability of an error in base calling. The percentage of bases > Q30 is averaged across the entire run. The quality scores for v2 chemistry > 80% bases higher than Q30 at 2 × 150 bp. The assembly of the reads was run to Genome Reference Consortium Human Build 37 (GRCh37) which is the human reference genome (version hg19). Image processing and Variant Call Format (VCF) file generation were further analyzed, we then annotated these variants using Illumina variant interpreter. Each variant is linked to numerical identifier in Catalogue of Somatic Mutations in Cancer (COSMIC) database. The likely impact of amino acid changes was determined with In Silico Predictions (Sift & PolyPhen) and Functional Analysis through Hidden Markov Models (v2.3) (FATHMM) prediction. The variants were categorized as benign or pathogenic according to ClinVar database. Mutations with low depth, which indicate ≤ 50× depths, mutations with ≤ 5% variant allele frequency, variants quality if < 80% and finally, variant that did not found in COSMIC database were filtered out.

### Microsatellite instability analysis

We assessed the microsatellite instability (MSI) status using 3 primer sequences (BAT25, BAT26, and NR27) according to manufacturer instructions with PCR products were analyzed on Agilent 2100 Bioanalyzer system as previously described. Tumor DNA was compared to that of healthy control with peaks present in the tumor that were not found in the normal subjects indicated instability of a marker. Patients with no varied markers were considered as microsatellite stable (MSS). Patients with only one varied microsatellite marker were considered as microsatellite instability-low (MSI-L) and those with ≥ 2 varied markers were classified as microsatellite instability-high (MSI-HI) [15].

## Results

### Patients’ characteristics

Eight breast cancer female patients were enrolled in the study and their clinico-pathological features at diagnosis including stages, hormonal receptor status, and molecular subtypes were shown in Table 1. Only one patient had metastatic BC at first presentation and she was 45 years old, 5 patients were proven to be metastatic BC during therapy and 2 patients had early non-metastatic BC. The histological subtype of all patients was invasive carcinoma of no specific type with median age of 41 years (range, 29–48 years).

**Table 1:** Characteristics of breast cancer patients

Patient’s characteristics	Total No. = 8 No. (%)
Age (years)	
Mean ± SD	40 ± 7.01
Median	41
Range	29-48
Stage at diagnosis	
II	2/8 (25%)
III	5/8 (62.5%)
IV	1/8 (12.5%)
Immunohistochemistry ER status	
Positive	5/8 (62.5%)
Negative	3/8 (37.5%)
PR status	
Positive	4/8 (50%)
Negative	4/8 (50%)
HER2 neu	
Positive	1/8 (12.5%)
Negative	7/8 (87.5%)
KI 67	
High	4/8 (50%)
Low	1/8 (12.5%)
Unknown	3/8 (37.5%)
Molecular subtype	
Luminal A	1/8 (12.5%)
Luminal B	4/8 (50%)
Triple negative disease	3/8 (37.5%)
Metastasis	
Metastatic:	6/8 (75%)
Liver	4/6 (66.7%)
Bone	3/6 (50%)
Lung	4/6 (66.7%)
Brain	0
Local recurrence	1/6 (16.7%)
Early disease, non metastatic:	2/8 (25%)
Microsatellite instability (MSI) status	
Microsatellite stable (MSS)	2/8 (25%)
Microsatellite instability-low (MSI-L)	3/8 (37.5%)
Microsatellite instability-high (MSI-HI)	3/8 (37.5%)

### Activating mutations’ results

Regarding mutational analysis, the variant allele frequency (VAF) was used to differentiate germline from somatic variants. A germline variant is identified with a 50% (heterozygous) or100% (homozygous) VAF. Acquired variant is present with a lower VAF as it is not found in all cells. Other factors may also contribute to VAF such as technical issues (polymerase chain reaction/amplification bias) can skew VAF. Also, somatic mutations may occur with VAF of 50% if the number of malignant cells in the analyzed sample is high. Finally, genetic features affect the VAF. VAF for each identified variant in each patient is shown in Table 2.

**Table 2:** Variant allele frequency (VAF) for each identified variant in each patient

Variant	No. of patients	P1	P2	P3	P4	P5	P6	P7	P8
		Metastatic	Metastatic	Metastatic	Metastatic	Metastatic	Early disease, non metastatic	Early disease, non metastatic	Metastatic
FLT3 Substitution—intronic	7	0.508	0.995	1	0.459		0.473	0.477	1
SMARCB1 Substitution—intronic	1	0.449							
FGFR3 p.(Thr653=) Substitution–coding silent	8	0.995	1	1	1	0.991	0.996	0.998	0.478
APC p.(Thr1493=) Substitution–coding silent	7	0.515	0.496	0.998	0.998	1	0.509		0.998
RET p.(Ser904=) Substitution–coding silent	2	0.473							0.489
RET p.(Leu769=) Substitution–coding silent	8	0.524	0.527	0.999	0.514	1	0.994	0.998	0.515
IDH1 p.(Gly105=) Substitution–coding silent	1	0.497							
HRAS p.(His27=) Substitution–coding silent	4			0.504		0.522	0.513	0.488	
EGFR p.(Gln787=) Substitution–coding silent	6	0.996	0.477	0.507	0.494		1		0.477
MET p.(Ile377=) Substitution–coding silent	1		0.484						
PDGFRA p.(Val824=) Substitution–coding silent	3			0.47	0.495				0.491
PDGFRA p.(Pro577=) Substitution–coding silent	1	0.522							
PDGFRA p.(Pro567=) Substitution–coding silent	8	0.997	0.469	0.999	0.998	1	1	0.998	0.998
STK11 p.(Tyr272=) Substitution–coding silent	1				0.522				
TP53 p.(Pro72Arg) Substitution—Missense	6	0.998	0.993	0.488			0.521	0.522	0.493
PIK3CA p.(Ile391Met) Substitution—Missense	2		0.505	0.555					
KDR p.(Gln472His) Substitution—Missense	2		0.573	0.138					
ATM p.(Phe858Leu) Substitution—Missense	1	0.465							
APC p.(Ile1307Lys) Substitution—Missense	1				0.518				
APC p.(Glu1317Gln) Substitution—Missense	1					0.504			
FGFR3 p.(Ala719Thr) Substitution—Missense	1			0.481					

**Fig. 1:**
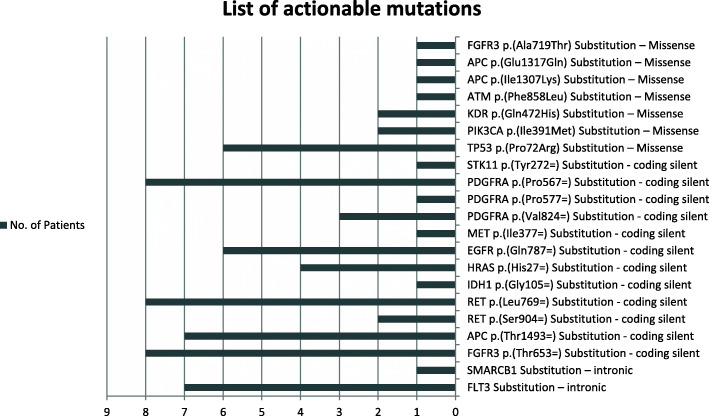
List of actionable mutations in studied breast cancer patients

Initial filtering yielded 42 variants as shown in Table 3. By searching about these variants in the COSMIC database version 92, we found that 13 variants did not found in COSMIC database, 6 variants are classified as a non-coding variant in COSMIC, because it was annotated in the intron of a transcript and 2 variants have been found as SNP. As a result, these variants were eliminated from our further discussion. The remaining 21 variants showed different activating mutations: 7 substitution–missense, 12 substitution–coding silent, and 2 substitution–intronic as shown in Fig. 1. These 21 activating mutations were found in 15 genes. Regarding ClinVar database, out of 21 variants there were 14 benign variants, 3 variants with conflicting interpretations of pathogenicity, 3 variants not reported, and 1 drug response variant.

**Table 3:** List of actionable mutations detected in 8 Egyptian breast cancer patients:

Gene	Variant	Consequence	Chr	Exons	dbSNP ID	HGVSc	HGVSp	No. of patients	In silico predictions		FATHMM prediction	ClinVar	COSMIC ID	MSI status *(no. of pts)*
									Sift	PolyPhen				
PIK3CA	SNV A>G	Substitution—Missense	3	7/21	rs2230461	c.1173A>G	p.(Ile391Met)	2	Tolerated (0.53)	Benign (0.015)	Not applicable	Benign	COSM328028 COSM5019247	MSI-HI MSI-L
	SNV A>G	Intron	3		rs3729674	c.352+40A>G		3				Uncertain significance	COSN26959779 COSN26959780	MSI-HI (2) MSI-L
KDR	SNV A>T	Substitution—Missense	4	11/30	rs1870377	c.1416A>T	p.(Gln472His)	2	Tolerated (0.16)	Benign (0.01)	Neutral (score 0.07)	Not reported	COSM149673	MSI-HI MSI-L
	SNV G>A	Intron	4		rs7692791	c.798+54G>A		8				Not reported	COSN8870412 COSN8870413	MSS (2) MSI-HI (3) MSI-L (3)
	SNV C>A	Intron	4		rs10006115	c.3849-24C>A		2				Not reported	COSN20494026	MSI-HI (2)
	INDEL	Intron	4		rs869246746 rs3214870 rs397772062	c.2615-37dupC		2				Not reported	COSN17154192	MSI-HI MSI-L
ATM	SNV T>C	Substitution—Missense	11	17/63	rs1800056	c.2572T>C	p.(Phe858Leu)	1	Tolerated (0.15)	Benign (0.105)	Pathogenic (score 0.73)	Conflicting interpretations of pathogenicity	COSM21826 COSM6493972	MSS
TP53	SNV C>G	Substitution—Miss ense	17	4/11	rs1042522	c.215C>G	p.(Pro72Arg)	6	Tolerated (0.57)	Benign (0.045)	Neutral (score 0.36)	drug response	COSM250061 COSM3766191	MSS (2) MSI-HI (2) MSI-L (2)
RET	SNV C>G	Substitution–coding silent	10	15/20	rs1800863	c.2712C>G	p.(Ser904=)	2			Neutral (score 0.27)	Benign	COSM3751779 COSM3751780	MSS MSI-L
	SNV G>T	Substitution–coding silent	10	13/20	rs1800861	c.2307G>T	p.(Leu769=)	8			Pathogenic (score 0.79)	Benign	COSM4418405 COSM4418406	MSS (2) MSI-HI (3) MSI-L (3)
IDH1	SNV C>T	Substitution–coding silent	2	4/10	rs11554137	c.315C>T	p.(Gly105=)	1			Pathogenic (score 0.85)	Benign	COSM1741220	MSS
HRAS	SNV T>C	Substitution–coding silent	11	2/6	rs12628	c.81T>C	p.(His27=)	4			Neutral (score 0.07)	Benign	COSM249860 COSM3752426	MSS MSI-L MSI-HI (2)
APC	SNV T>A	Substitution—Missense	5	16/16	rs1801155	c.3920T>A	p.(Ile1307Lys)	1	Tolerated (0.7)	Benign (0.005)	Pathogenic (score 0.94)	Conflicting interpretations of pathogenicity, risk factor​	COSM26697	MSI-HI
	SNV G>C	Substitution—Missense	5	16/16	rs1801166	c.3949G>C	p.(Glu1317Gln)	1	Tolerated (0.06)	Benign (0.005)	Pathogenic (score 0.98)	Conflicting interpretations of pathogenicity	COSM19099	MSI-L
	SNV G>A	Substitution–coding silent	5	16/16	rs41115	c.4479G>A	p.(Thr1493=)	7			Neutral (score 0.46)	Benign	COSM3760869	MSS MSI-HI (3) MSI-L(3)
EGFR	SNV G>A	Substitution–coding silent	7	20/28	rs1050171	c.2361G>A	p.(Gln787=)	6			Pathogenic (score 0.95)	Benign	COSM1451600	MSS MSI-HI (3) MSI-L(2)
MET	SNV C>T	Substitution–coding silent	7	2/21	rs28444388	c.1131C>T	p.(Ile377=)	1			Neutral (score 0.29)	Benign	COSM5020205	MSI-HI
FLT3	SNV T>C	Substitution—intronic	13		rs2491231	c.1310-3T>C		7			Neutral (score 0.02)	Not reported	COSM3999060	MSS (2) MSI-HI (3) MSI-L (2)
SMARCB1	SNV G>A	Substitution—intronic	22		rs5030613	c.1119-41G>A		1			Neutral (score 0.03)	Benign	COSM1090 COSN17135779	MSS
PDGFRA	SNV C>T	Substitution–coding silent	4	18/23	rs2228230	c.2472C>T	p.(Val824=)	3			Pathogenic (score 0.88)	Benign	COSM22413	MSI-L (2) MSI-HI
	SNV G>A	Substitution–coding silent	4	12/23	rs55830582	c.1731G>A	p.(Pro577=)	1			Neutral (score 0.03)	Benign	COSM9494799	MSS
	SNV A>G	Substitution–coding silent	4	12/23	rs1873778	c.1701A>G	p.(Pro567=)	8			Neutral (score 0.02)	Benign	COSM7410554	MSS (2) MSI-HI (3) MSI-L (3)
FGFR3	SNV G>A	Substitution—Missense	4	16/18	rs17882190	c.2155G>A	p.(Ala719Thr)	1	Tolerated (0.15)	Benign (0.06)	Pathogenic (score 0.89)	Likely Benign	COSM9067744	MSI-L
	SNV G>A	Substitution–coding silent	4	14/18	rs7688609	c.1959G>A	p.(Thr653=)	8			Pathogenic (score 0.70)	Not reported	COSM7410552	MSS (2) MSI-HI (3) MSI-L (3)
	SNV C>T	Splice region Intron	4		rs1227316073	c.1965+6C>T		1				Not reported		MSI-L
FGFR2	SNV T>C	Intron	10		rs145303463	c.939+11T>C						Benign/Likely benign​		MSS
ABL1	SNV G>A	Synonymous Variant	9	4/11	rs2229069	c.777G>A	p.(Thr259=)	1				Benign	–	MSI-HI
NOTCH1	SNV C>T	Synonymous Variant	9	34/34	rs372760677	c.7390C>T	p.(Leu2464=)	2				Benign/Likely benign	–	MSS MSI-HI
ERBB4	SNV T>C	Synonymous Variant	2	9/28	rs148466450	c.1024T>C	p.(Leu342=)	1				Likely Benign	–	MSI-HI
	SNV A>G	Intron	2		rs839541	c.421+58A>G		4				Not reported	COSN19690034 COSN27007111	MSS MSI-HI (2) MSI-L
	INDEL	Splice region Intron	2		rs748883732	c.884-8_884-7delTT						Not reported		MSI-L
	INDEL	Splice region Intron	2		rs67894136 rs397987661	c.884-7delT		8				Not reported	–	MSS (2) MSI-HI (3) MSI-L (3)
STK11	SNV C>T	Substitution–coding silent	19	6/10	rs9282859	c.816C>T	p.(Tyr272=)	1			Pathogenic(score 0.86)	Benign	COSM29005	MSI-HI
	SNV T>C	Intron	19		rs2075606	c.465-51T>C		3				Uncertain significance​	COSN6666958	MSS MSI-HI MSI-L
ALK	SNV G>T	Intron	2		rs3738868	c.3836+27G>T		2				Not reported		MSI-HI (2)
AKT1	SNV G>A	Intron	14		rs61761180	c.46-28G>A		1				Not reported		MSI-HI
BRAF	SNV T>G	Intron	7		rs6959000	c.1315-18T>G						Benign		MSI-L
RB1	SNV C>T	Intron	13		rs765434764	c.1389+39C>T						Not reported		
NPM1	INDEL	Intron	5		rs397792554 rs34323200	c.847-5delT		8				Not reported		MSS (2) MSI-HI (3) MSI-L (3)
CSF1R	MNV	3-prime UTR	5	22/22	rs386693509	c.*35_*36delCAinsTC		8				Uncertain significance​		MSS (2) MSI-HI (3) MSI-L (3)

### Substitution–missense mutations

Tumor protein TP53 (*TP53*) gene mutations were substitution–missense mutations and were detected in 6 patients. All were p.(Pro72Arg) drug response variant which occur as a result of substitution at c.215C>G. Phosphatidylinositol 4,5-bisphosphate 3-kinase catalytic subunit alpha (*PIK3CA*) gene mutation was detected in 2 patients, both were substitution–missense mutations p.(Ile391Met) as a result of substitution at c.1173A>G and classified as benign variant in ClinVar database. Kinase insert domain receptor (*KDR*) gene is a vascular endothelial growth factor receptor 2 (*VEGFR2*) gene and its mutation was found in 2 patients. They were substitution–missense mutations p.(Gln472His) that result from substitution at c.1416A>T and such variant not reported in ClinVar database. Only one patient revealed ataxia telangiectasia mutated (*ATM*) gene mutation. It was substitution–missense mutation p.(Phe858Leu) resulting from substitution at c.2572T>C and reported in ClinVar database as a variant of conflicting interpretations of pathogenicity. However, it was predicted to be pathogenic variant according to FATHMM prediction score. One patient showed substitution–missense mutation in FGFR3 gene (c.2155G>A). It is reported in ClinVar database as likely benign variant, but it is pathogenic at FATHMM prediction. Two patients showed substitution–missense mutations in APC Regulator of WNT signaling pathway (*APC*) gene. One patient showed p.(Ile1307Lys) variant as a result of substitution at c.3920T>A and classified in ClinVar database as a variant of conflicting interpretations of pathogenicity, risk factor. The other patient was p.(Glu1317Gln) resulting from substitution at c.3949G>C and considered in ClinVar database as a variant of conflicting interpretations of pathogenicity. These 2 *APC* variants were predicted to be pathogenic variants according to FATHMM prediction score.

### Substitution–coding silent mutations

Seven patients revealed substitution–coding silent mutations in *APC* gene which were benign p.(Thr1493=) variant resulting from substitution at c.4479G>A. Two benign substitution–coding silent mutations were detected in Ret Proto-Oncogene (*RET*) gene. The first one was p.(Ser904=) variant as a result of c.2712C>G and was found in 2 patients. The other one was p.(Leu769=) resulting from c.2307G>T and was detected in 8 patients. Again substitution–coding silent mutations were detected in isocitrate dehydrogenase (NADP(+)) 1 (*IDH1*) gene in only 1 sample, HRas Proto-Oncogene, GTPase (*HRAS*) gene in 4 samples, epidermal growth factor receptor (*EGFR*) gene in 6 samples, MET Proto-Oncogene, receptor tyrosine kinase (*MET*) gene in only 1 sample, fibroblast growth factor receptor 3 (*FGFR3*) gene in 8 samples, and Serine/threonine kinase 11 (STK11) gene in only 1 sample. These variants were p.(Gly105=), p.(His27=), p.(Gln787=), p.(Ile377=), p.(Thr653=), and p.(Tyr272=), respectively resulting from substitution at c.315C>T, c.81T>C, c.2361G>A, c.1131C>T, c.1959G>A, and c.816C>T, respectively. Platelet-derived growth factor receptor A (PDGFRA) gene revealed 3 substitution–coding silent mutations p.(Val824=), p.(Pro577=), and p.(Pro567=) that result from c.2472C>T, c.1731G>A and c.1701A>G, respectively.

### Substitution–intronic mutations

Finally, 2 substitution–intronic were found in Fms related receptor tyrosine kinase 3 (*FLT3*) gene in 7 samples and SWI/SNF related, matrix associated, actin dependent regulator of chromatin, subfamily B, member 1 (*SMARCB1*) gene in only 1 sample as a result of substitution at c.1310-3T>C and c.1119-41G>A, respectively.

### Microsatellite instability status

**Fig. 2:**
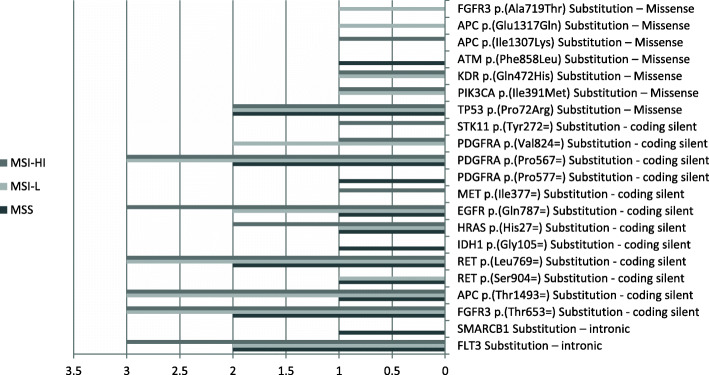
Relation between MSI status and different studied activating mutations

Regarding microsatellite instability (MSI) status, 2/8 (25%) patients were MSS, 3/8 (37.5%) patients were MSI-L, and 3/8 (37.5%) patients were MSI-HI. Correlation between MSI status and different studied somatic mutations was shown in Fig. 2.

## Discussion

The application of liquid biopsy in solid tumors affords a useful and secure method to identify the existence of actionable driver mutations, to monitor response to therapy, to discover recent recurrence, to aid the radiological imaging in post-treatment surveillance, and to predict cancer therapy outcomes [16]. The crucial intent of precision medicine in cancer patients is to adjust clinical management according to targeted molecular profiling. Next-generation sequencing is increasingly used in identification of somatic mutations in each cancer patient and such information can direct treatment decisions [17]. Here, we identify the activating mutations in liquid biopsy of Egyptian breast cancer patients using targeted next generation sequencing technology. Also, we detect the microsatellite instability (MSI) status using BAT25, BAT26, and NR27 markers which are tested on the Bioanalyzer 2100 system.

*TP53* gene is a tumor suppressor gene that control DNA repair and apoptosis mechanisms. TP53 mutation is frequently observed in BC and it accounts for nearly 30% of all BC cases. In our study, all *TP53* mutations were p.(Pro72Arg) drug response variant and found in 6 out of 8 patients (75%). It was described previously that *TP53* p.(Pro72Arg) variant denotes BC susceptibility [18]. On the other hand, some reports found that *TP53* p.(Pro72Arg) revealed no significant association with BC risk [19, 20]. In addition, it was noted that TP53 mutation with the R72 variant was significantly correlated with poor prognosis in BC females. Therefore, *TP53* codon 72 might be a powerful anticipating marker for chemotherapeutic response in BC [21].

PIK3CA mutations are detected in ~ 30–40% of BC patients and lead to alpha isoform (p110α) of the phosphatidylinositol 3-kinase (PI3K) hyperactivation [22]. We found that 2 out of our 8 patients (25%) revealed *PIK3CA* p.(Ile391Met) mutation. These 2 patients were metastatic BC patients, one of them was stage III and the other was stage IV at diagnosis. Nassar et al. 2020 found PIK3CA I391M polymorphism in 7 patients (15.2%) and revealed that it could be used as BC tumorigenesis marker [23]. Ahmadi et al. 2017 revealed that PIK3CA I391M (rs2230461 A>G) genetic polymorphism is not correlated with breast cancer risk. Yet, he found significant differences in stage III BC patients compared to control group which may be a molecular sign that reveals the PIK3CA rs2230461 can be associated with the starting of breast cancer cells invasion [24]. Finally, in a pre-clinical experiment, trastuzumab efficacy was appraised against many HER2-positive cell lines. ZR-75-30 cell line expressing PIK3CA I391M was associated with same sensitivity to trastuzumab in comparison to SK-BR-3 cell line expressing PIK3CA wild-type, at all concentrations tested (0–10 μg/mL) [25].

Kinase insert domain receptor (*KDR*) gene is a vascular endothelial growth factor receptor 2 (*VEGFR2*) gene. The PI3K/Pten pathway is one of the downstream signalings affected by KDR activation and most commonly altered in breast cancer [26]. In our study, *KDR* p.(Gln472His) mutation was found in the same 2 patients who revealed *PIK3CA* p.(Ile391Met) mutation.

Two patients showed *APC* substitution–missense mutations, one patient showed p.(Ile1307Lys) variant, and the other patient was p.(Glu1317Gln). *APC* p.(Ile1307Lys) was reported previously as risk factor for susceptibility to BC [27]. The *APC* p. Glu1317Gln variant is known as conflicting interpretation of pathogenicity in ClinVar, although it may have pathogenic effect and was detected in one pancreatic cancer patient and one breast cancer patient from different families [28].

Finally, only one patient revealed ATM p.(Phe858Leu) gene mutation. In US, this missense mutation occurred at ~ 2% frequency and was associated with a significant increased BC risk [29].

Microsatellite instability (MSI) is remarkably low in BC, in spite of extensive clinical expectations that various patients might be responsive to immune checkpoint inhibitors [30]. However in our study, 2/8 (25%) patients were MSS, 3/8 (37.5%) patients were MSI-L, and 3/8 (37.5%) patients were MSI-HI. A relatively larger cohort is needed for further and precise analysis of these genetic markers and MSI status in Egyptian BC patients.

## Conclusion

It is essential to use and validate minimally invasive liquid biopsy for activating mutations detection by next-generation sequencing especially in patients with inoperable disease or bone metastasis. This work should be extended with larger patient series with comparison of genetic mutations in liquid-based versus tissue-based biopsy and longer follow up period.

## Data Availability

The datasets used and/or analyzed during the current study are available from the corresponding author on reasonable request.

## Abbreviations

APC: APC Regulator of WNT signaling pathway
ATM: Ataxia telangiectasia mutated
BC: Breast cancer
COSMIC: Catalogue of Somatic Mutations in Cancer
ctNA: Circulating tumor nucleic acids
EGFR: Epidermal growth factor receptor
FATHMM: Functional Analysis through Hidden Markov Models (v2.3)
FFPE: Formalin-fixed paraffin-embedded
FLT3: Fms-related receptor tyrosine kinase 3
gDNA: Genomic DNA
GRCh37: Genome Reference Consortium Human Build 37
HRAS: HRas Proto-Oncogene, GTPase
HS: High sensitivity
IDC: Invasive duct carcinoma
IDH1: Isocitrate dehydrogenase (NADP(+)) 1
IRB: Institutional Review Board
KDR: Kinase insert domain receptor
MET: MET proto-oncogene, receptor tyrosine kinase
MSI: Microsatellite instability
MSI-HI: Microsatellite instability-high
MSI-L: Microsatellite instability- low
MSS: Microsatellite stable
NCRP: National Cancer Registry Program
NGS: Next-generation sequencing
PDGFRA: Platelet-derived growth factor receptor alpha
PIK3CA: Phosphatidylinositol 4,5-bisphosphate 3-kinase catalytic subunit alpha
Q-score: Quality score
RET: Ret proto-oncogene
SMARCB1: SWI/SNF Related, Matrix Associated, Actin Dependent Regulator of Chromatin Subfamily B, member 1
STK11: Serine/threonine kinase 11
TP53: Tumor protein TP53
VAF: Variant allele frequency
VCF: Variant Call Format
VEGFR2: Vascular endothelial growth factor receptor 2
